# Quantitative proteomics characterization of cancer biomarkers and treatment

**DOI:** 10.1016/j.omto.2021.04.006

**Published:** 2021-04-20

**Authors:** Xiao-Li Yang, Yi Shi, Dan-Dan Zhang, Rui Xin, Jing Deng, Ting-Miao Wu, Hui-Min Wang, Pei-Yao Wang, Ji-Bin Liu, Wen Li, Yu-Shui Ma, Da Fu

**Affiliations:** 1 National Engineering Laboratory for Deep Process of Rice and Byproducts, College of Food Science and Engineering, Central South University of Forestry and Technology, Changsha 410004, Hunan, China; 2 Cancer Institute, Nantong Tumor Hospital, Affiliated Tumor Hospital of Nantong University, Nantong 226631, China; 3 Central Laboratory for Medical Research, Shanghai Tenth People’s Hospital, Tongji University School of Medicine, Shanghai 200072, China; 4 Department of Radiology, The Fourth Affiliated Hospital of Anhui Medical University, Hefei 230012, China

**Keywords:** quantitative proteomics, cancer, biomarker, diagnostic marker, therapeutic target

## Abstract

Cancer accounted for 16% of all death worldwide in 2018. Significant progress has been made in understanding tumor occurrence, progression, diagnosis, treatment, and prognosis at the molecular level. However, genomics changes cannot truly reflect the state of protein activity in the body due to the poor correlation between genes and proteins. Quantitative proteomics, capable of quantifying the relatively different protein abundance in cancer patients, has been increasingly adopted in cancer research. Quantitative proteomics has great application potentials, including cancer diagnosis, personalized therapeutic drug selection, real-time therapeutic effects and toxicity evaluation, prognosis and drug resistance evaluation, and new therapeutic target discovery. In this review, the development, testing samples, and detection methods of quantitative proteomics are introduced. The biomarkers identified by quantitative proteomics for clinical diagnosis, prognosis, and drug resistance are reviewed. The challenges and prospects of quantitative proteomics for personalized medicine are also discussed.

## Graphical abstract

**Figure:**
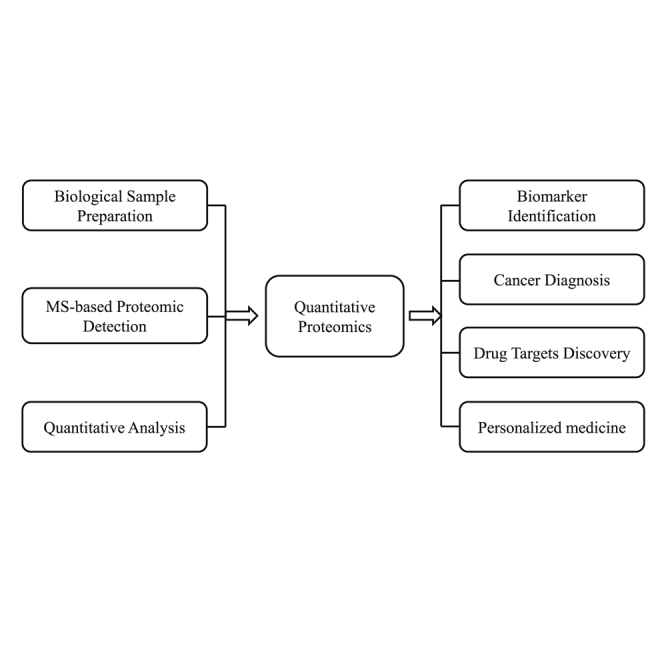

## Teaser

Yang et al. introduce the development, samples tested, and detection methods of quantitative proteomics. Biomarkers for clinical diagnosis, prognosis, and drug resistance identified by quantitative proteomics are reviewed. The challenges and prospects of quantitative proteomics for personalized medicine are also discussed.

## Introduction

Cancer accounted for 16% of global deaths according to the 2018 American Association for Cancer Research (AACR) Cancer Progress Report. Global cancer incidence will continue to increase with the aging of the population. By 2040, the number of new cancer cases is expected to reach 27.5 million worldwide, and the number of deaths is expected to reach 16.3 million. The Cancer Genome Atlas (TCGA) database has published the genomic landscapes of 33 tumor types from 11,000 patients, which could help to promote the understanding of tumor occurrence, progression, diagnosis, treatment, and prognosis at the molecular level.^1^, ^2^, ^3^, ^4^, ^5^ As the direct executor of life activities, protein participates in almost all life processes, such as heredity, development, reproduction, material and energy metabolism, and stress.^6^ However, the correlation between genomics changes and protein abundance is very poor, especially for low-abundance proteins.^7^ Therefore, proteomics could be the bridge between genome information and functional proteins that helps to further the understanding of cancer.^8^^,^^9^

Proteomics is based on the protein composition and changing of cells, tissues, or organisms. Proteomics studies the characteristics of proteins on a large scale, including protein expression levels, post-translational modifications (PTMs), and protein-protein interactions, to gain a comprehensive understanding of disease occurrence, cell metabolism, and other processes at the protein level.^10^, ^11^, ^12^ Quantitative proteomics provides comprehensive information on the protein interactions, signal pathways, and biomarkers of human disease by detecting the relative changes in protein abundance in diseased tissue samples.^13^^,^^14^ In this review, we discuss the application of quantitative proteomics in cancer research and the discovery of tumor biomarkers, as well as its potential significance in early clinical diagnosis, prognosis, and targeted therapy.

## Development of quantitative proteomics

**Figure 1:**
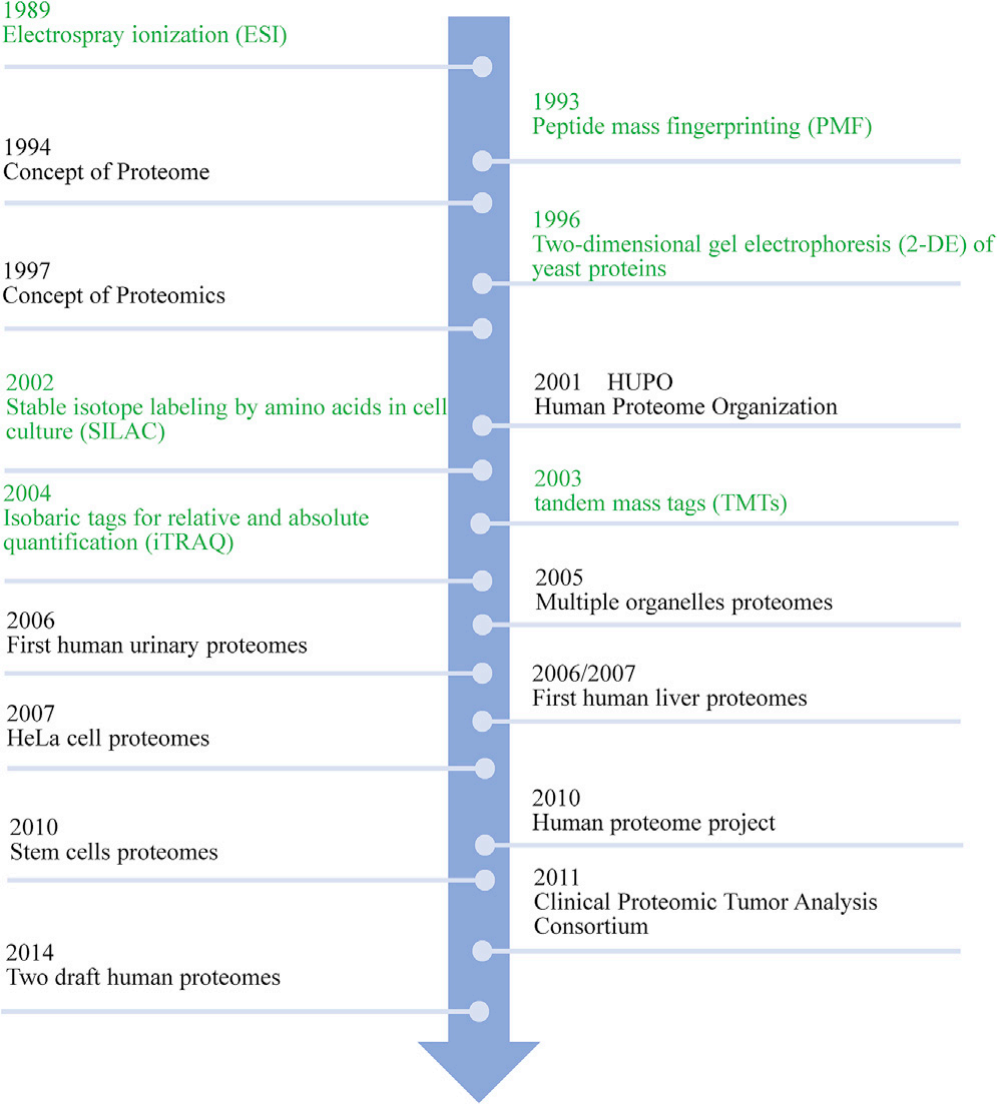
The development of quantitative proteomics Green indicates technical MS advances; black indicates MS-identified human proteomes.

The concept of the proteome was first put forward by Australian scientist Mark Wilkins in 1994, and the concept of proteomics was put forward in 1997 as a science that studies the composition and changes of proteins in cells, tissues, or organs.^15^ In 2001, the International Human Proteome Organization (HUPO) officially announced the promotion of proteomics research. In the past 20 years, proteomics technology has improved continuously, which has enabled the application of quantitative analysis methods in proteomics.^4^^,^^16^, ^17^, ^18^, ^19^, ^20^ In 2014, *Nature* published two papers on human proteome for the first time.^21^^,^^22^ The application of proteomics has extensively promoted the progress of natural science research (Figure 1).

**Figure 2:**
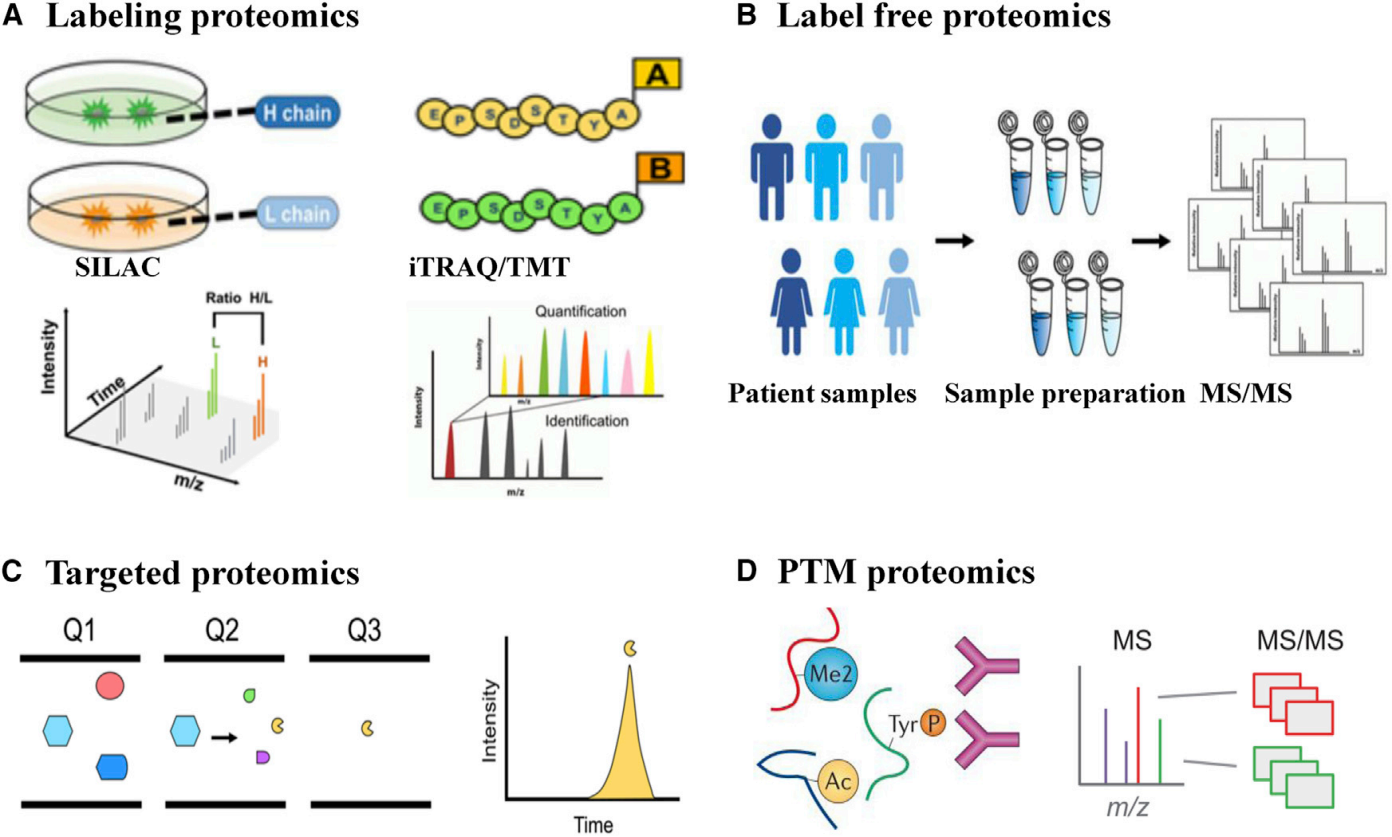
A comparison of detection methods used in quantitative proteomics (A) Labeling proteomics: SILAC is used for cell lines, iTRAQ/TMT is used for labeling *in vitro*, and MS/MS spectra are assigned to peptides for identification and quantitation. (B) Label-free proteomics is used to quantify the protein expression across different samples. (C) Targeted proteomics, selected from three quadrupoles (Q1, Q2, Q3), is suitable for identifying and quantitating target peptides within complex mixtures. (D) PTM proteomics: using antibody-based immunoprecipitation (IP) to enrich peptides containing modifications (phosphorylation [P], dimethyl [Me2], or acetylation [Ac]), LC-MS/MS is used for peptide identification and quantitation.

Currently, there are four main quantitative proteomics methods, that is, labeling, label-free, targeted, and PTM, widely used in cancer research (Figure 2). Stable isotope labeling by amino acids in cell culture (SILAC) technology, isobaric tags for relative and absolute quantification (iTRAQ) technology, and tandem mass tags (TMTs) technology are the main methods used for the labeling quantitative proteomics.^23^, ^24^, ^25^, ^26^ SILAC technology is suitable for analyzing living cells in culture with accurate quantification and good repeatability.^27^ SILAC removes the false positives in protein-interaction studies, reveals the large-scale kinetics of proteomes, and directly uncovers the important points in the cellular signaling pathways as a quantitative phosphoproteomics technology. The triple-label SILAC proteomic profiles have been used to reveal the deregulation of key cell cycle regulators in long intergenic non-coding RNA-nucleotide metabolism regulator (lincNMR)-depleted cells, such as the key 2′-deoxynucleoside 5′-triphosphate (dNTP) synthesizing enzymes RRM2, TYMS, and TK1, which implicated lincNMR in regulating nucleotide metabolism.^28^ The iTRAQ/TMT technology has high sensitivity, high throughput, and good reproducibility.^29^^,^^30^ Keller et al.^31^ reported that secretome analysis using iTRAQ proteomics revealed the caspase-1-mediated secretion of other leaderless proteins with known or unknown extracellular functions. Without labeling processing, label-free quantification is simple to conduct, but it requires high stability and repeatability of experimental operations. It is suitable for large-scale quantitative comparison and experimental design that cannot be realized with labeling quantification.^32^ Wepr et al.^33^ presented a label-free mass spectrometry-based strategy for the absolute quantification of protein complex components isolated through affinity purification and quantitatively analyzed the interaction stoichiometries in the human protein phosphatase 2A network.

**Figure 3:**
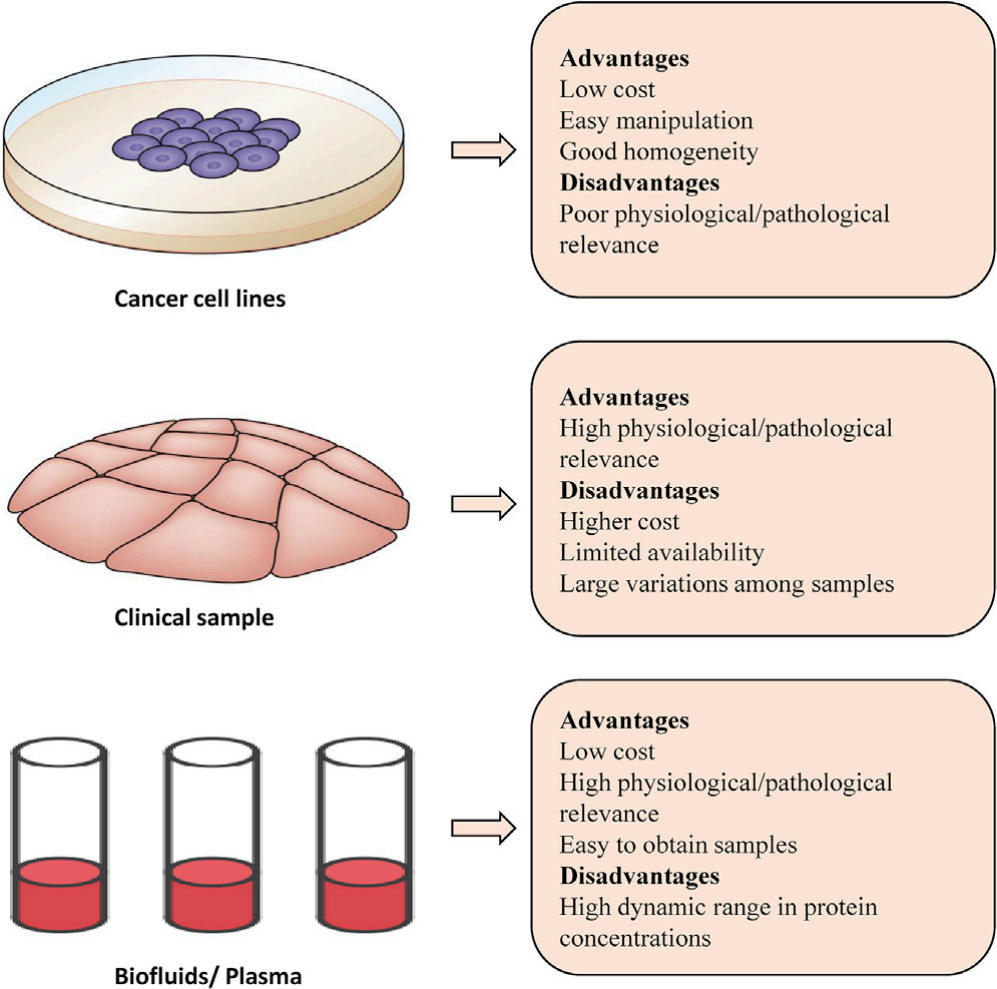
A comparison of the biological samples used in quantitative proteomics There are three samples for quantitative proteomics analysis, as shown on the left. Each type of sample has its advantages and disadvantages, as shown on the right.

Targeted quantitative proteomics is essentially a mass spectrum scanning mode based on the selection of specific target protein ion and product ion pairs.^34^, ^35^, ^36^ Targeted quantitative proteomics could detect the relative or absolute quantities of various target proteins in complex samples (Table 1). PTM is an important component of protein activity regulation.^39^, ^40^, ^41^, ^42^ Phosphorylation modification is the most common and most important PTM regulating the protein kinase and other protein activities.^43^ Quantitative phosphoproteomics is widely used for proteomic stratification and drug target identification. Jiang et al.^43^ adopted proteomic and phosphoproteomic profiling and characterized 110 paired tumor and non-tumor tissues of clinical early-stage hepatocellular carcinoma (HCC) related to hepatitis B virus (HBV) infection. The quantitative proteomics data highlighted the heterogeneity in early stage HCC. Many analytical methods of proteomics have been developed for different samples, including cell lines, clinical samples, and body fluids.^13^^,^^37^^,^^38^ Each type of sample has advantages and disadvantages (Figure 3). The choice of sample type depends on the purpose of the research.

**Table 1:** A comparison of detection methods for quantitative proteomics

	Methods of label	Applicable samples	Clinical samples	Advantages	Disadvantages	Application	Ref.
SILAC	*in vivo* metabolic incorporation of lysine or arginine	tissue culture cells	no	high sensitivity	high cost limited to living samples	biomarker screening in cell lines	^27^^,^^28^
				high accuracy			
				high repeatability			
				closely reflect the state of samples			
				high sensitivity			
iTRAQ / TMT	*in vitro* N terminus and lysine side chains of peptide	non-living samples	yes	compare 2–10 samples in parallel	poor to low-abundance proteins	biomarker screening	29, 30, 31
				high coverage			
				high throughput			
				high accuracy			
Label-free proteomics	no	non-living samples	yes	low cost	poor stability and repeatability	biomarker screening	^32^^,^^33^
				simple manipulation			
				not limited by samples			
				high throughput			
				closely reflect the state of samples			
				high sensitivity			
Targeted proteomics	no	non-living samples	yes	high accuracy	poor to higher protein complexity and complex analysis	intestinal flora screening	34, 35, 36
				high repeatability			
				wider dynamic range			
PTM proteomics	no	non-living samples	yes	closely reflect the state of samples	high requirements for peptide enrichment	biomarker and drug target screening	^13^^,^^37^^,^^38^
				kinase target screening

## Quantitative proteomics classification of tumor subtype

In clinical practice, there is an urgent need for the early detection of cancer and the differentiation of tumor subtypes to improve the existing treatment. Proteome-informed classification could distinguish the clinical features of early-stage non-smoker lung adenocarcinoma.^44^^,^^45^ Mass spectrometry-based proteomic profiling could classify the pan-cancer molecular subtypes of 532 cancers.^46^ Quantitative proteomics could identify and quantify the specific signaling pathways from the tumor tissues and corresponding para-tumor tissues of 24 patients at different stages of triple-negative breast cancer (TNBC).^47^ Quantitative proteomics could be used for the accurate classification of TNBC subtypes.^48^ Furthermore, the sub-network identified through quantitative phosphoproteomics was highly correlated with clinically identified breast cancer subtypes.^49^, ^50^, ^51^ SWATH/DIA-MS (state-of-the-art sequential windowed acquisition of all theoretical fragment ion/data-independent acquisition mass spectrometry) presented a promising complement for the stable classification of ovarian cancer subtypes.^52^^,^^53^ Quantitative proteomics of reverse-phase protein array (RPPA) could be used to classify diffuse large B cell lymphoma.^54^, ^55^, ^56^

## Identifying potential biomarkers with quantitative proteomics

**Figure 4:**
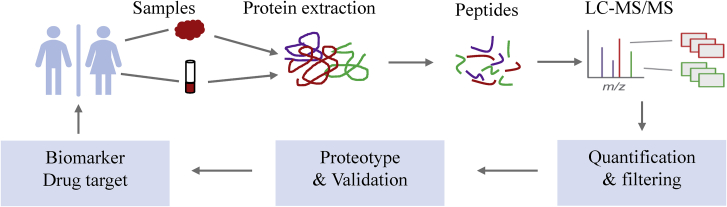
Integrated view of LC-MS/MS proteomics workflow for cancer biomarker discovery Step 1: cancer tissues and adjacent tissues for protein extraction are prepared. Step 2: the proteins are enzymatically digested into peptides. Step 3: the peptides are analyzed with LC-MS/MS. Step 4: databases are mapped to peptides and proteins through quantification and filtering. Step 5: proteotype-like PPI interactomes are generated by further data validation. Step 6: candidate biomarkers and drug targets are identified. Step 7: after functional verification, biomarkers and drug targets are recommended to clinical medicine.

With the development of mass spectrometry, quantitative proteomics has become an important method to discover tumor biomarkers. Increasing amounts of tumor biomarkers have been discovered by quantitative proteomics.^57^, ^58^, ^59^ Samples from tumor tissue and paired adjacent tissue or patients and healthy people were prepared, digested into peptides, and then analyzed with liquid chromatography-tandem mass spectrometry (LC-MS/MS). After quantification and filtration, tumor biomarkers were identified (Figure 4).

**Figure 5:**
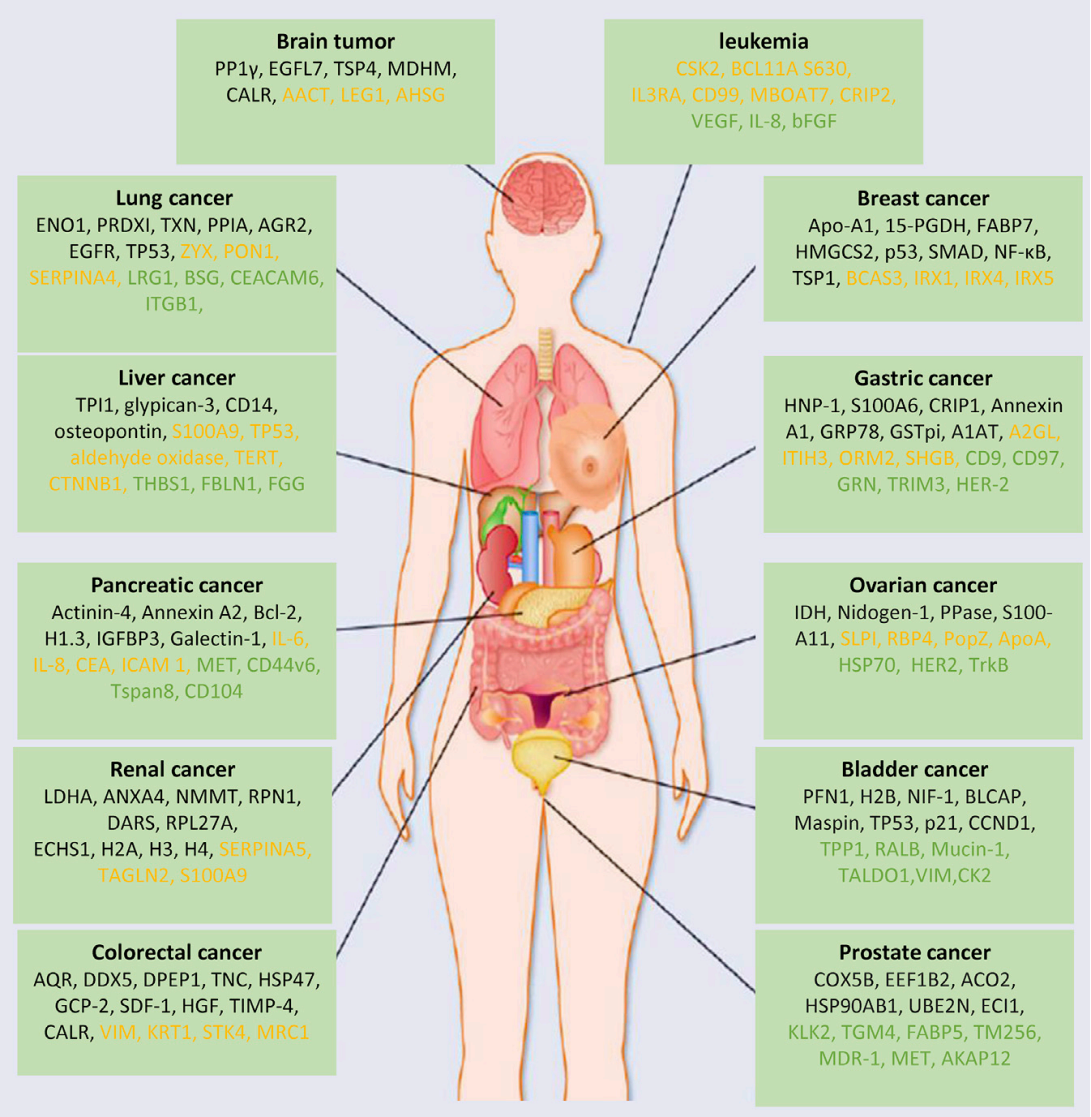
Quantitative proteomics adopted in the discovery of various cancer biomarkers Many biomarkers for different types of cancer are identified through quantitative proteomics. Biomarkers were found from cancer tissue (black), plasma/serum (orange), and exosome (green).

The comprehensive classification of lung adenocarcinoma provided bioinformatics resources for clinical treatment, drug development, and precision medicine.^60^, ^61^, ^62^ Quantitative analysis of control, HBV, cirrhotic, and HCC tissue showed CD14 as a promising biomarker.^63^ The DIA quantitative proteomics analysis of 10 paired tumor and non-tumor samples verified three oxidative phosphorylation biomarkers (UQCRQ, NDUFB7, and UQCRC2) in gastric cancer.^37^ By the quantitative analysis of tumor tissues against normal adjacent tissues (NATs), AQR, DDX5, DPEP1, and TNC were identified as biomarkers in colorectal cancer. Through the proteomics approach, triosephosphate isomerase 1 (TPI1) was identified as a biomarker for predicting the recurrence of intrahepatic cholangiocarcinoma.^64^ Quantitative proteomics is increasingly adopted for identifying biomarkers of early pancreatic cancer, such as actinin-4, annexin A2, Bcl-2, H1.3, IGFBP2, IGFBP3, and galectin-1 (Figure 5).^65^, ^66^, ^67^, ^68^, ^69^, ^70^, ^71^

## Discovering drug targets with quantitative proteomics

Quantitative proteomics is a promising tool for revealing the molecular mechanisms of drug action.^58^^,^^63^, ^64^, ^65^, ^66^, ^67^, ^68^, ^69^, ^70^, ^71^, ^72^, ^73^, ^74^, ^75^ Proteomics drug maps have greatly promoted the discovery of drug targets. After the quantitative analysis for 10,000 proteins and 55,000 phosphorylation sites (p-sites) from 125 cancer cell lines, the proteome activity landscapes were obtained. Adenylate kinase isoenzyme 1 (AK1) was discovered as a promising drug target for acute myeloid leukemia patients.^76^ The anaplastic lymphoma kinase (ALK) inhibitor ceritinib was found to be capable of modulating the protein-trafficking and degradation-related process of autophagy after the quantitative analysis of five lung cancer cell lines in response to more than 50 drugs.^77^ The proto-oncogene serine/threonine-protein kinase PIM3 has been widely used as a drug target. Quantitative phosphoproteomics revealed that PIM3 activated RhoA to promote migration and invasion of hepatoma cells.^78^ By comprehensive phosphoproteomics characterization of 110 tumors and 101 matched NATs, three candidate drug targets were identified for lung adenocarcinoma (LUAD), including SOS1 inhibition in KRAS mutant, PTPN11/Shp2 inhibition in both ALK fusion and EGFR mutant tumors, and STK11 mutation in neutrophil degranulation.^62^ Quantitative proteomics was adopted to characterize 200 paired EGFR-positive and EGFR-negative glioma tissues of all pathological types, and EGF-like domain multiple 7 (EGFL7) was identified as a potential diagnostic biomarker and therapeutic target.^79^

## Discovering drug resistance biomarkers with quantitative proteomics

Drug resistance and recurrence are the main obstacles to the long-term survival of cancer patients. It is crucial to understand the mechanisms and identify the biomarkers of drug resistance. Quantitative proteomics could help to identify the proteins related to drug resistance.^80^, ^81^, ^82^

Tamoxifen resistance is one of the unsolved problems in breast cancer treatment. Through proteomics analysis of tumor tissues from tamoxifen therapy-sensitive and tamoxifen therapy-resistant breast cancer patients, high expression of ectonucleotide pyrophosphatase/phosphodiesterase-1 (ENPP1) and extracellular matrix metalloproteinase inducer (EMMPRIN) were found relevant to tamoxifen resistance. However, low expression of eukaryotic translation initiation factor 3 subunit 6/E (EIF3E) and guanine nucleotide-binding protein β subunit 4 (GNB4) were relevant to tamoxifen resistance.^83^ Moreover, EMMPRIN-negative tumors were more sensitive to neoadjuvant chemotherapy in bladder cancer (BC).^84^ Using label-free quantitative proteomics analysis of trastuzumab-resistant MKN45/R cells and parental MKN45 human gastric cancer cells, WNT signaling was identified as a potential target in trastuzumab-resistant cancer. Quantitative proteomics analysis of the anti-HCC efficacy of dihydroartemisinin (DHA) combined with sorafenib may help to understand the related molecular mechanism of anti-HCC.^85^ The drug resistance of ovarian cancer cell lines was evaluated with iTRAQ LC-MS/MS, and 28 biomarkers that might lead to cisplatin resistance were identified.^86^ Radio resistance biomarkers in several cancers, such as breast cancer, prostate cancer, and lung cancer, were identified with MS-based proteomics approaches.^87^

Protein kinases are primary molecular drug targets, and phosphorylation regulation is a key mechanism in cancer drug resistance. Through integrated proteomics and phosphoproteomics analysis of cisplatin-sensitive (T24S) and cisplatin-resistant (T24R) T24 human BC cell lines, CDK2 was identified as a potential chemoresistance biomarker in BC.^88^ Through phosphoproteomics analysis of lapatinib-sensitive (SKBR3) and lapatinib-resistant (SKBR3-LR) breast cell lines, p21-activated kinase 2 (PAK2) was identified as an effective therapeutic target to overcome acquired lapatinib resistance in HER2-positive breast cancer.^89^ The success in identifying cancer drug resistance biomarkers could help to develop biomarker-guided targeted therapy.

## The application of quantitative proteomics in clinical diagnosis and treatment

Liquid biopsy is increasingly recognized as a promising non-invasive identification method of clinical biomarkers. Many studies have shown that exosomes, i.e., 40- to 100-nm vesicles containing nucleic acids, proteins, and lipids, could be used as tumor biomarkers.^90^, ^91^, ^92^ Various biomarkers for different types of cancer have been identified with exosome proteomics (Figure 5). Thrombospondin-1 (THBS1), fibulin-1 (FBLN1), and fibrinogen gamma chain (FGG) were identified as clinical biomarkers for liver cancer.^93^, ^94^, ^95^ Leucine-rich alpha-2-glycoprotein 1 (LRG1), basigin (BSG), carcinoembryonic antigen-related cell adhesion molecule 6 (CEACAM6), and integrin beta-1 (ITGB1) were identified as clinical biomarkers for lung cancer.^96^, ^97^, ^98^, ^99^ Plasma or serum is an important component for liquid biopsy. The plasma protein level of HSP90β was validated as a potential prognostic biomarker in LUAD after a comprehensive proteomics analysis of 103 cases in China.^60^ The serum amyloid A protein was identified as a biomarker for renal cancer by comparing 119 patients with clear cell RCC and 69 healthy controls. BCAS3, IRX1, IRX4, and IRX5 were identified in breast cancer plasma samples through label-free quantitative proteomics.^100^, ^101^, ^102^, ^103^ S100P and aldehyde oxidase were identified as potential liver cancer biomarkers from human serum through quantitative proteomics (iTRAQ).^104^ SOD2 was identified as a potential salivary biomarker in liver cancer through iTRAQ-based proteomics.^105^

## Conclusions

With the development of mass spectrometry technology, quantitative proteomics has been widely applied for studying cancer mechanisms. Many biomarkers of different cancers identified with quantitative proteomics could help in the early diagnosis, prognosis, and drug resistance analysis.^106^^,^^107^ Three types of samples, including cell lines, clinical samples, and body fluids, are used in quantitative proteomics research. Clinical samples and body fluids are widely used in cancer research. Several biomarkers of 12 types of cancers identified from clinical samples and body fluids are listed in Figure 5. Liquid biopsy is increasingly recognized as a promising non-invasive identification method of clinical biomarkers. Many tumor-related biomarkers have been found in serum, urine, saliva, and exosomes.^100^, ^101^, ^102^, ^103^ Due to high protein complexity and wide dynamic range, quantitative proteomics for liquid biopsy face significant challenges. Future research may focus on developing mass spectrometry technology with wider coverage and dynamic range.

A series of proteomics technologies have been developed for the comprehensive understanding of cancer occurrence and development mechanisms, including PTM proteomics, spatiotemporal proteomics, single-cell proteomics, and multi-omics. Since the functional diversity of proteins is achieved through PTMs, many protein kinases have been identified as drug targets through quantitative phosphorylation proteomics.^108^, ^109^, ^110^ Spatiotemporal proteomics allows the identification of proteins that change subcellular localization under different experimental conditions using quantitative proteomics.^111^ As a topic of frequent discussion in the past decade, single-cell proteomics evaluates the heterogeneity and rare types of cells based on cell types and the state of a single cell.^112^^,^^113^ Multi-omics approaches have become promising in the study of human diseases.^60^, ^61^, ^62^ HSP90β was identified as a potential prognostic biomarker for lung cancer through integrative analysis of proteome, phosphoproteome, transcriptome, and whole-exome sequencing data.^60^ A complicated regulatory map of the SLC2A2 gene with 16 candidate enhancers was identified for HCC by coupling transcriptome and proteome.^114^ The effective integration of all of these technologies eventually promotes accurate diagnosis and personalized medicine.
